# Constructing a Nurse-led Cardiovascular Disease Intervention in Rural Ghana: A Qualitative Analysis

**DOI:** 10.5334/aogh.3379

**Published:** 2021-11-30

**Authors:** Ethan P. Wood, Katherine L. Garvey, Raymond Aborigo, Edith Dambayi, Denis Awuni, Allison P. Squires, Elizabeth F. Jackson, James F. Phillips, Abraham R. Oduro, David J. Heller

**Affiliations:** 1 Arnhold Institute for Global Health, Icahn School of Medicine at Mount Sinai, New York, NY, United States of America; 2 Navrongo Health Research Center, Navrongo, Ghana; 3 New York University Rory Meyers College of Nursing, New York, NY, United States of America; 4 Columbia Mailman School of Public Health, New York, NY, United States of America

## Abstract

**Background:**

Cardiovascular disease (CVD) is a growing burden in low- and middle-income countries. Ghana seeks to address this problem by task-shifting CVD diagnosis and management to nurses. The Community-Based Health Planning and Services (CHPS) initiative offers maternal and pediatric health care throughout Ghana but faces barriers to providing CVD care. We employed in-depth interviews to identify solutions to constraints in CVD care to develop a nurse-led CVD intervention in two districts of Ghana’s Upper East Region.

**Objective:**

This study sought to identify non–physician-led interventions for the screening and treatment of cardiovascular disease to incorporate into Ghana’s current primary health care structure.

**Methods:**

Using a qualitative descriptive design, we conducted 31 semistructured interviews of community health officers (CHOs) and supervising subdistrict officers (SDOs) at CHPS community facilities. Summative content analysis revealed the most common intervention ideas and endorsements by the participants.

**Findings:**

Providers endorsed three interventions: increasing community CVD knowledge and engagement, increasing nonphysician prescribing abilities, and ensuring provider access to medical and transportation equipment. Providers suggested community leaders and volunteers should convey CVD knowledge, marshaling established gathering practices to educate communities and formulate action plans. Providers requested lectures paired with experiential learning to improve their prescribing confidence. Providers recommended revising reimbursement and equipment procurement processes for expediting access to necessary supplies.

**Conclusions:**

Frontline CHPS primary care providers believe CVD care is feasible. They recommended a three-pronged intervention that combines community outreach, provider training, and logistical support, thereby expanding task-shifting beyond hypertension to include other CVD risk factors. This model could be replicable elsewhere.

## Introduction

Cardiovascular disease (CVD) is a growing global public health challenge, causing 17.3 million deaths worldwide in 2015 [1]. Hypertension, a key risk factor for CVD, afflicts approximately 1 billion people worldwide, and low- and middle-income countries (LMIC) bear two-thirds of the global burden of hypertension [2]. LMICs also a face significant burden from other CVD risk factors; for instance, they comprise 80% of all tobacco smokers globally [3].

The prevalence of CVD and its risk factors is high in sub-Saharan Africa (SSA), where changes in diet toward more energy-rich food and falling levels of physical activity have contributed to a sharp rise in noncommunicable diseases (NCD), such as CVD, obesity, and diabetes mellitus [4]. Pooled data suggests a prevalence of hypertension of 30% to 31% in Sub-Saharan Africa [5], including 46% of adults over the age of 25 [6] and 57% of those over 50 [5]. However, public awareness of disease prevalence is low. Research conducted in West, East, and South Africa shows 39% of men and 54% of women with known hypertension are unaware of their hypertensive status [7]. Within rural and urban Ghana, CVD risk factor prevalence is increasing. Tobacco use in Ghana is 8.7% among men and 0.4% among women, with rural populations disproportionately affected [8]. The incidence of overweight and obesity within Ghana are 25.4% and 17.1%, respectively [9]. Mortality due to hypertension and cardiometabolic diseases is rising in both rural and urban settings, while areas with fewer health care providers report higher NCD rates [7,10].

Lack of CVD understanding hinders efforts to address its growing prevalence. Previous research revealed inconsistent awareness of key risk factors, such as diet, smoking, and obesity, in the development of CVD in Ghana [11]. Rural areas, such as the Upper East Region of northern Ghana, are particularly vulnerable due to a scarcity of medical professionals compounded by pervasive poverty associated with a subsistence agricultural economy and low literacy rates [12]. Surveillance data from the Upper East community of Navrongo, accordingly, reveals an adult hypertension prevalence of 24.5% in persons aged 40–60 years, with awareness under 35% [7].

To address the growing burden of CVD, health care systems with physician shortages are relying on nonphysicians to increase access to CVD care, a strategy known as task-shifting [13,14]. With proper training, nonphysician health workers can manage hypertension as effectively as physicians [15]. Task-shifting is also economically beneficial, ultimately lowering health systems’ overhead costs [16]. In recent years, experts have validated complex protocols for nurse-led control of CVD risk factors, including the World Health Organization’s HEARTS model [17], but how to integrate these programs with existing initiatives in low-resource settings remains unclear.

Ghana’s commitment to task-shifting for improving access to primary care within its resource-constrained health system is not new—but the focus has not been CVD. Policies promoting community-based health care date back to Ghana’s endorsement of the 1978 Alma Ata Declaration and subsequent efforts in the 1990s to fulfill this commitment [18]. In 1994, the Ghana Ministry of Health launched a pilot of community primary health care in three communities [19]. Based on this operational model, a plausibility experiment was launched in 1996 to gauge the impact on childhood survival [20]. The success of this trial led to a series of Ghana Health Service–sponsored replication studies that culminated in a scaling-up initiative known as Community-Based Health Planning and Services (CHPS) [21]. CHPS is designed to deliver primary care, chiefly maternal and child health care, in resource-poor settings in Ghana [20,21]. It deploys nurses from subdistrict and district clinics to live and work in satellite community health compounds (CHCs), facilitating the provision of door-to-door health screening and care. Initial pilots of the program showed a 50% reduction of child mortality in 3 years, leading to expansion and implementation across the country [20]. Today, each nurse is managed by a subdistrict officer (SDO), typically a medical assistant of a subdistrict health center and occasionally a midwife. Each SDO supervises a CHPS zone, which comprises three to five CHCs where community health officers (CHO) reside. National monitoring of the CHPS expansion suggests the goal of achieving full national coverage of CHPS service operations is feasible [22].

While CHPS has been remarkably effective in improving access to reproductive and child health services, it was not designed to screen for and treat CVD. Previous research identified key barriers CHPS nurses and their supervisors face in providing CVD screening and care to their communities, which include lack of training and relevant medications and limited community awareness of hypertension and CVD [23]. Other recent studies have pioneered CHPS care for hypertension but not global CVD risk [13]. Because multiple risk factors contribute to the development of CVD, including hypertension, hyperlipidemia, diets low in fruits and vegetables, physical inactivity, depression, and tobacco use, a joint intervention to impact all these factors is essential.

This study builds upon current CVD prevention work by identifying solutions and pathways for an integrated CVD care initiative based on the HEARTS protocol within CHPS compounds. Specifically, we use nonphysician health care provider knowledge and insight in the Upper East Region of Ghana to identify feasible changes to the current CHPS framework to allow CVD to be better identified and treated within rural communities.

## Methods

### Design

A qualitative descriptive design structured this study. Qualitative descriptive designs are the most flexible of all qualitative approaches to research in that they seek to describe a phenomenon through the descriptive experiences of the participants [24,25,26]. In this case, we sought participant input about their experiences with a variety of interventions that could potentially adapt CHPS to facilitate CVD management. The Institutional Review Boards of the Icahn School of Medicine at Mount Sinai (IRB-16-01189) and the Navrongo Health Research Centre (IRB-250) approved this study.

### Study Site

The study was conducted in the Kassena-Nankana West and Kassena-Nankana Municipal districts around the town of Navrongo in the Upper East Region of Ghana, localities where communities have been reached by research operations of the Navrongo Health Research Centre ever since a trial of vitamin A supplementation was launched in 1989 [27,28]. This area of Ghana is among the poorest in the country. Pervasive poverty and low educational attainment complicate the provision of effective primary health care [12,29]. Data on cardiovascular disease risk in this region include a rise in adult hypertension prevalence from 19.1% to 24.5% from 2008 to 2017 [6,30]. In the Navrongo area, the prevalence of overweight or obesity is 7.2% among men and 18.4% among women [31]; as of 2018, only 5.5% of the population of the Kassena-Nankana districts had elevated LDL cholesterol, but 60.3% had low HDL cholesterol [32].

### Sampling

Study participants were health care workers within CHCs in the Kassena-Nankana districts of Ghana’s Upper East Region. They were either CHOs, nurses provided with 18 months of training for conducting basic ambulatory, preventive, and promotional health care services in the community where their CHC is based, or SDOs, CHPS clinics’ administrative directors based in subdistrict health centers. SDOs often work as midwives at their clinics as well. Nurses and midwives are distinct professions in Ghana, although some are cross-trained for both roles.

### Data Collection

A semistructured interview guide (1) gauged participants’ beliefs and responses to previously determined barriers to providing CVD screening and treatment and (2) elicited participants’ attitudes and beliefs about interventions on the individual, CHC, and national level that will best allow for CHPS to screen for and treat CVD and its risk factors. Interviews were conducted on-site at CHCs during the months of June and July 2018 over a course of 6 weeks. Interviews were conducted in English—the language of nursing and midwifery education in Ghana—after obtaining written consent from each participant. Durations ranged from 24 to 63 minutes. This study was conducted as a joint endeavor of the Navrongo Health Research Centre (NHRC), Columbia University, and the Mount Sinai School of Medicine. D.J.H. planned the project protocol in collaboration with J.F.P. and A.R.O. Interviews were conducted by E.P.W. and K.L.G., both American medical students, with one additional interview conducted by E.D. E.D., D.A., and other NHRC staff supervised E.P.W. and K.L.G. at all interviews. This approach was intended to convey the research study to participants as a partnership cocreated and overseen by the NHRC, which has an extensive prior history of collaborative research with both the CHPS health program and the Navrongo community as a whole and employs chiefly local and national staff to underscore its mission as a Ghanaian health development institute. Moreover, NHRC staff arranged all interviews, answered any procedural or ethical questions about the research and its aims, and confirmed respondents’ facility in the English language. Both medical students also completed cultural competency training at their home institution prior to their arrival in Ghana. Sessions were audio recorded and then transcribed by the research team (E.P.W., K.L.G.). We then comparatively cross-checked each team member’s transcript to reconcile inconsistencies and ensure accuracy [33]. Data were deidentified upon completion of transcription.

### Analysis

To analyze the interviews, we used summative content analysis to determine the interventions most often mentioned or described by participants, followed by an iterative generation of other themes and categories that emerged from the analysis. Summative content analysis specifically accounts for the frequency with which participants mention something, in this case interventions, within a specific context [34]. This had the added benefit of examining the data in its entirety before identifying specific interventions.

We created codes for both barriers to CVD care and corresponding interventions through an iterative process wherein team members separately reviewed transcripts and met to compare identified codes for interventions. Participants would then determine which existing codes best represented the data and create new additional codes as needed to classify uncoded interventions. This ensured a shared understanding of each code’s and intervention’s meaning and increased the consistency of code application. After a consistent set of codes describing various interventions emerged, all transcripts were coded. NVivo was used for coding and analysis (NVivo 11, Melbourne, Australia) [35].

## Results

A total of 21 CHOs and 10 SDOs participated in the study. Participants were recruited through a network of CHCs described elsewhere [22]. Participants identified three potential interventions for adaptation in the interviews that could render CVD care through CHPS.

### Community Engagement and Education Regarding CVD and Treatment

This intervention seeks to promote understanding related to CVD prevention and treatment within communities and improve CHPS health care professionals’ community relationships.

Several participants suggested collaborative meetings between CHPS leadership and community elders as the first step to develop stronger community ties for CVD engagement. Participants described community leaders as influential figures with the ability to convey the importance of CVD screening to reluctant members of the community. One SDO suggested, “The community leaders, when they talk to their people, they understand, they listen to them more than we, the health workers.”

Participants also cited community volunteers as important allies for improving community understanding of CVD. Because the CHPS program operates across all of Ghana, CHOs working in the Upper East Region commonly come from another region of the country. Consequently, they face challenges effectively communicating and building rapport with community members. As a mitigation measure, some CHOs use community volunteers as cultural liaisons. Several CHOs suggested leveraging community volunteers to aid in community education efforts. One CHO described this role of community volunteers, saying, “They should at least add the community volunteers…. [T]hey even know those people more than us. So if we are able to train them and they are able to train the community, they will be the best.”

Participants also suggested using preexisting community practices, specifically community gatherings called *durbars*, to improve awareness of CVD risk factors and introduce screening measures. Durbars aim to share news and facilitate important discussions within Kassena-Nankana communities. Almost all participants endorsed durbars as the best place for CVD education and screening, granted that increased financial support was offered to offset durbar-related costs. One CHO suggested, “We can organize durbars to create awareness…. [W]e can organize the durbar and do the screening there.”

Finally, participants emphasized that visual aids and educational materials would allow more fruitful discussions about CVD. As one CHO explained, “I think with this one, when they see the pictures they’ll feel like ‘wow, so having BP and heart disease, this is what it looks like compared to normal,’ so at the end of the day you’re able to do two things. You talk to the person, yes, and you also show pictures and the person is able to see what is happening. Then at the end of the day I think that the truth will help the person be serious and get more insight of what you are telling the person.”

CHOs most often requested visual aids depicting the various effects of untreated CVD. Participants believed such resources would promote understanding of CVD among persons not literate in English and clearly convey the consequences of not receiving CVD care. Several participants also suggested that local radio stations discuss CVD to educate community members who are intimidated or reluctant to engage with CHPS health care providers. As one CHO explained, “I think the education we should bring to the lower level or to the local dialect so that they can understand by going to the radio station to educate them, and let the people be aware that there is something.”

### Access Barriers to CVD Screening and Treatment

This intervention seeks to address material barriers to providing CVD care. SDOs and CHOs cited a lack of material supplies and timely health insurance reimbursements as the greatest access challenges.

Participants specifically identified motorbikes and blood pressure cuffs as the most essential CVD care supplies. Catchment areas of CHPS zones often incorporate a large area of land with small communities scattered throughout. CHOs rely on motorbikes to conduct home visits. Unanimously, CHOs cited a lack of functioning motorbikes as the greatest barrier to providing CVD care. One SDO explained, “The logistics, that’s the motorbikes and fuel when you need to do the work, that is my biggest problem…. [T]hat is hampering us most.”

Participants also stated a lack of functioning blood pressure cuffs as a barrier to CVD care. Most CHPS clinics have only one or two blood pressure cuffs. Commonly, all midwives, community health nurses, and CHOs within a single clinic share one blood pressure cuff. Consequently, CHOs are unable to bring blood pressure cuffs to home visits.

Participants offered several ideas for how to better supply CHPS compounds with motorbikes and blood pressure cuffs. SDOs requested freedom to negotiate with health insurance and equipment providers within the Ghana Health Service (GHS). SDOs speculated direct communication with equipment suppliers within GHS might expedite shipping and processing time for new equipment. One SDO said, “We were now thinking … our leaders (SDOs) should also liaise with the health insurance, then they will be paying in time, if we meet then they pay so that we’ll get money to buy this BP apparatus. At times you order from the medical store and they (blood pressure cuffs) are not there.”

Participants also encouraged the GHS to seek philanthropic funds for equipment and to emphasize providing on-site accommodations for staff to improve their ability to consistently render services. They cited the contributions of the Korean International Cooperation Agency (KOICA), which has provided funding to improve primary care in rural Ghana. Participants praised these efforts and suggested GHS pursue additional philanthropic funds to meet their equipment needs. One CHO, referring to KOICA’s work, said the following: “And they make sure you have a portion to maintain your motorbike throughout the quarter. And then to organize an activity. So we don’t lack fuel, we don’t lack anything like that. So they came and they actually helped us.”

Finally, participants emphasized delays in health insurance reimbursement from GHS as a major barrier to CVD care. Currently, providers at CHPS clinics submit reimbursement paperwork after providing services to patients insured by GHS. They then use reimbursement funds to purchase necessary supplies for ongoing care at CHCs. Unfortunately, SDOs reported significant delays in receiving reimbursement funds. Some participants said they wait more than a year for reimbursements. SDOs called for higher-level policy changes for how the GHS manages reimbursement. They suggested simplifying the required documentation for services rendered and making administrative staff more accessible. Reducing the amount of documentation required for services rendered would minimize paperwork errors, which inevitably delay reimbursement.

### Workforce CVD Knowledge and Autonomy

This intervention addresses a lack of CVD knowledge among CHOs and SDOs and increases workforce autonomy. CHOs and SDOs unanimously endorsed inadequate knowledge in CVD screening and treatment and the inability to prescribe hypertensive medications as hinderances to their current work. One CHO explained, “You can’t go into detail because you yourself don’t have the knowledge about it (CVD)…. So people may have it but they don’t know they have it because we don’t have much information about it.”

All participants expressed interest in receiving training on CVD screening and treatment. Most commonly, participants suggested opportunities to learn both the “theoretical” and practical aspects of CVD care. Several participants suggested training should begin with workshops organized at the subdistrict level so CHOs and SDOs receive a “refresher” course on CVD in a group setting. Participants preferred small group teaching for its personalized, stress-free environment.

Additionally, CHOs and SDOs requested medical professionals who already treat CVD to be instructors. One SDO envisioned each workshop including a doctor to coach CHOs on the nuances of CVD screening and treatment: “Give them a workshop where you can attach yourself to a medical doctor … and we can now discuss about the type of drugs that can be given at this level.”

Another SDO explained the importance of current providers’ insight into recognizing the signs and symptoms of CVD: “They have to organize trainings for us…. And then we invite people who are into treating cardiac diseases, so they will train them and let them understand when they get these signs and symptoms of this condition.”

In addition to workshops, participants requested opportunities to implement newly acquired CVD knowledge under direct supervision. Participants requested sessions where doctors accompany and observe CHOs on home visits and in the clinic as an opportunity for immediate feedback. One SDO explained, “And you can also choose to come around, when we are doing consultation, then you see how we do it, then on the spot you correct.”

Currently, the capacity of CHOs to treat CVD is significantly hindered by their inability to prescribe antihypertensive medications. The current classification system of the CHPS program excludes antihypertensive medications from being prescribed or reimbursed by insurance. Several participants were frustrated that they cannot do anything beyond measuring blood pressure when trying to treat CVD in their communities. Many community members cannot easily travel to larger towns with hospitals that offer antihypertensive medications. One SDO stated, “Because here we don’t deal with the heart diseases directly, when they come and we suspect, we refer, so when they come back they tell us that because they would have to go to the hospital and buy a lot of drugs some still don’t have (the medications). So they quickly, silently go back to the house and wait for death.”

The majority of SDOs and CHOs expressed confidence in their ability to prescribe antihypertensive medications to CVD patients with proper training. One SDO stated, “Once it’s to help our people, I think we will not hesitate, we will embrace it and do it well.”

Finally, several SDOs emphasized workforce augmentation will be critical to a successful intervention, since CHCs are understaffed and already provide a variety of services.

## Discussion

We aimed to use health workers’ local knowledge and experiences to design a sustainable intervention to screen for and treat CVD risk factors in rural northern Ghana while addressing previously identified challenges to such care [23]. Specific practices emerged to address each of these barriers: increasing community engagement and knowledge of CVD through coordinated community gatherings and increasing community stakeholder participation; enhancing provider knowledge of CVD care and safe administration of antihypertensive medications by CHPS providers; and bolstering resource availability, including medical equipment, staff support, and transportation. In discussing these interventions, participants emphasized (1) an enthusiasm to adopt such interventions and expand their services and (2) the relative ease with which such adaptations could be introduced into the current CHPS framework, granted appropriate workforce augmentation. Participants stated the proposed CVD interventions are appropriate for the local cultural context in which they will be introduced. Additionally, rather than placing additional strain on the current CHPS structure, participants believed that the proposed interventions—if achieved—would improve access to equipment and transportation as well as communication and collaboration across different levels of the CHPS organizational structure.

Ample literature suggests task-shifting to nurses is a feasible CVD control strategy, even in fragile health care systems [36,37,38]. However, these interventions require training, materiel, and especially human resources, and their scope of CVD care and efficacy in achieving it depend on how these resources are applied. For example, studies in Cameroon and Nigeria demonstrate that nonphysicians can intervene to lower blood pressure, and a nurse-led hypertension intervention in the Ashanti region of Ghana was positively received by nonphysician providers [39,40,41]. But the first of these interventions struggled with frequent reassignment of scant nurse providers [39]. Conversely, the second benefited from pharmacists adjusting hypertension medications in lieu of overworked physicians [40]. And prior work in Ghana demonstrated that CHPS nurses can identify and refer patients with hypertension to nearby clinics [13], but personnel time, equipment, and transportation remain as scale-up threats [41].

Our results suggest solutions to these challenges, such as strengthening leadership engagement with the proposed task-shifting intervention and widening the local availability of health care resources [37]. The high degree of readiness for change suggests these interventions could viably surmount those barriers in addressing not just blood pressure but CVD overall. If created in collaboration with community members and their leaders, this intervention could address not only classic CVD risk factors, such as tobacco use or hypertension, but also prevalent comorbid local risk factors, such as alcohol use disorder, depression, and other mental illness [23], which cause and are caused by CVD [42,43,44]. The prevalence of depression in persons in Ghana with hypertension, for example, may be as high as 41.7% [45]. With the prudent use of existing local human resources, such as CHPS community health volunteers and pharmacists, to aid nurses in treating and counseling patients on CVD risk control on-site at CHPS compounds, such an intervention could avoid disrupting the current operation of the CHPS health network and minimize added overhead cost to the system by creating new referral clinics.

Our study has several notable limitations. All qualitative investigation carries a risk of biases associated with disparities in power and privilege between interviewee and interviewer, especially if the two come from different cultural or linguistic backgrounds. Our use of non-Ghanaian interviewers increased the risk of such bias. We mitigated that risk through the joint design of all aspects of our study between local and global partners—including survey design, data analysis, and results reporting—as well as the use of an interview partnership team. However, such bias cannot be eliminated, nor its extent fully quantified. More broadly, this study’s qualitative nature precludes generalizability beyond the study’s setting, but the approach may prove useful for replication in sites with similar characteristics. All interviews were conducted in English because CHPS staff are fluent in this language, as it is used for all formal clinical training. However, many staff learned English only as a second language for professional purpose and speak a different language at home. This limitation may have limited the scope and specificity of some participants’ responses.

Additionally, this study emphasizes the perspective of CHOs and SDOs working within the CHPS system. However, CHPS has as complex, multitiered organizational structure, with district directors and district health management teams (DHMT) coordinating and supervising multiple SDOs. As such, a final limitation of this paper is a large-scale systems perspective of the current CHPS leadership is not represented within our participant pool. Further work should be done to clarify leadership and training responsibilities, care referrals, and acute care roles across these different management system roles to better enable future CVD interventions to succeed on a larger systems level.

## Conclusion

The health care workers interviewed in this study believe the current CHPS framework is readily adaptable for task-shifting for CVD screening and management at the community level. In conjunction with augmentation of the CHPS workforce, participants proposed a three-pronged intervention to enhance community knowledge of CVD care; increase provider knowledge of CVD management, thus permitting prescription of antihypertensive medications; and improve systems for the reliable procurement and supply of medical equipment and transportation. A pilot program implemented at a handful of select CHPS clinics could assess the operational feasibility, acceptability, and efficacy of this intervention. This work could, in turn, be leveraged for other chronic conditions, given the potential flexibility of the current CHPS structure to adapt to the rapidly shifting disease burden across rural Ghanaian communities.

## References

[1] WHO Updates on Cardiovascular Disease. Accessed March 29, 2021. https://www.who.int/cardiovascular_diseases/about_cvd/en/.

[2] Perkovic V, Huxley R, Wu Y, Prabhakaran D, MacMahon S. The burden of blood pressure-related disease: a neglected priority for global health. Hypertension. 2007; 50(6). DOI: 10.1161/HYPERTENSIONAHA.107.095497 17954719

[3] World Health Organization. WHO Global Report on Trends in Prevalence of Tobacco Smoking 2000–2025. 2nd ed. World Health Organization; 2018.

[4] Bawah AA, Houle B, Alam N, et al. The evolving demographic and health transition in four low- and middle-income countries: evidence from four sites in the INDEPTH network of longitudinal health and demographic surveillance systems. PLoS One. 2016; 11(6). DOI: 10.1371/journal.pone.0157281 PMC4909223 27304429

[5] Bosu WK, Reilly ST, Aheto JM, Zucchelli E. Hypertension in older adults in Africa: a systematic review and meta-analysis. PloS One. 2019; 14(4): e0214934. DOI: 10.1371/journal.pone.0214934 30951534 PMC6450645

[6] World Health Organization. A Global Brief on Hypertension: World Health Day 2013. World Health Organization; 2013.

[7] Gomez-Olive X, Ali S, Made F, et al. Regional and sex difference in the prevalence and awareness of hypertension: An H3Africa AWI-Gen study across 6 sites in sub-Saharan Africa. Global Heart. 2017; 12(2). DOI: 10.1016/j.gheart.2017.01.007 PMC5967381 28302553

[8] Owusu-Dabo E, Lewis S, McNeill A, Gilmore A, Britton J. Smoking uptake and prevalence in Ghana. Tobacco Control. 2009; 18(5). DOI: 10.1136/tc.2009.030635 PMC2745559 19581276

[9] Ofori-Asenso R, Agyeman A, Laar A, Boateng D. Overweight and obesity epidemic in Ghana—a systematic review and meta-analysis. BMC Public Health. 2016; 16(1). DOI: 10.1186/s12889-016-3901-4 PMC5148846 27938360

[10] Vedanthan R, Ray M, Fuster V, Magenheim E. Hypertension treatment rates and health care worker density: an analysis of worldwide data. Hypertension. 2019; 73: 594–601. DOI: 10.1161/HYPERTENSIONAHA.118.11995 30612489 PMC6374168

[11] Murray M, King C, Sorensen C, Bunick E, King R. Community awareness of stroke, hypertension and modifiable risk factors for cardiovascular disease in Nkonya-Wurupong, Ghana. J Public Health Africa. 2018; 9(2). DOI: 10.4081/jphia.2018.783 PMC6325415 30687476

[12] Adongo PB, Phillips JF, Kajihara B, Fayorsey C, Depuur C, Binka FN. Cultural factors constraining the introduction of family planning among the Kassena-Nankana of northern Ghana. Social Science & Medicine. 1997; 45(12). DOI: 10.1016/S0277-9536(97)00110-X 9447629

[13] Ogedegbe G, Plange-Rhule J, Gyamfi J, et al. Health insurance coverage with or without a nurse-led task shifting strategy for hypertension control: A pragmatic cluster randomized trial in Ghana. PLoS Medicine. 2018; 15(1). DOI: 10.1371/journal.pmed.1002561 PMC5929500 29715303

[14] Heller DJ, Kumar A, Kishore SP, Horowitz CR, Joshi R, Vedanthan R. Assessment of barriers and facilitators to the delivery of care for noncommunicable diseases by nonphysician health workers in low- and middle-income countries. JAMA Network Open. 2019; 2(12). DOI: 10.1001/jamanetworkopen.2019.16545 PMC6902752 31790570

[15] Gaziano TA, Abrahams-Gessel S, Denman CA, et al. An assessment of community health workers’ ability to screen for cardiovascular disease risk with a simple, non-invasive risk assessment instrument in Bangladesh, Guatemala, Mexico, and South Africa: An observational study. Lancet Global Health. 2015; 3(9). DOI: 10.1016/S2214-109X(15)00143-6 PMC4795807 26187361

[16] Joshi R, Alim M, Kengne AP, et al. Task shifting for non-communicable disease management in low and middle income countries: a systematic review. PLoS One. 2014; 9(8). DOI: 10.1371/journal.pone.0103754 PMC4133198 25121789

[17] HEARTS Technical Package. World Health Organization; 2016. Accessed March 29, 2021. http://www.who.int/cardiovascular_diseases/hearts/en/.

[18] Amonoo-Lartson R. Change in family health care policy: the Ghanaian experience. In: The Non-Physician and Family Health in Sub-Saharan Africa: Proceedings of a Conference. Pathfinder Fund; 1981: 108–115.

[19] Binka FN, Nazzar A, Phillips JF. The Navrongo community health and family planning project. Studies in Family Planning. 1995; 26(3). DOI: 10.2307/2137832 7570763

[20] Phillips JF, Bawah AA, Binka FN. Accelerating reproductive and child health programme impact with community-based services: The Navrongo experiment in Ghana. Bulletin of the World Health Organization. 2006; 84(12). DOI: 10.2471/BLT.06.030064 PMC2627578 17242830

[21] Nyonator FK, Awoonor-Williams JK, Phillips JF, Jones TC, Miller RA. The Ghana community-based health planning and services initiative for scaling up service delivery innovation. Health Policy Planning. 2005; 20(1). DOI: 10.1093/heapol/czi003 15689427

[22] Awoonor-Williams JK, Ayaga AB, Nyonator FK, et al. The Ghana essential health interventions program: a plausibility trial of the impact of health systems strengthening on maternal & child survival. BMC Health Services Research. 2016; 13. DOI: 10.1186/1472-6963-13-S2-S3 23819518 PMC3668206

[23] Haykin, L, Francke J, Abapali A, et al. Adapting a nurse-led primary care initiative to cardiovascular disease control in Ghana: a qualitative study. BMC Public Health. 2020; 20(1). DOI: 10.1186/s12889-020-08529-4 PMC7245779 32448243

[24] Doyle, L, McCabe, C, Keogh, B, Brady, A, McCann, M. An overview of the qualitative descriptive design within nursing research. Journal of Research in Nursing. 2019; 25(5). DOI: 10.1177/1744987119880234 PMC7932381 34394658

[25] Willis, DG, Sullivan-Bolyai, S, Knafl, K, Cohen, MG. Distinguishing features and similarities between descriptive phenomenological and qualitative description research. Western Journal of Nursing Research. 2016; 38(9). DOI: 10.1177/0193945916645499 27106878

[26] Sandelowski, M. What’s in a name? Qualitative description revisited. Research in Nursing & Health. 2009; 33(1). DOI: 10.1002/nur.20362 20014004

[27] Oduro A, Wak G, Azongo D, et al. Profile of the Navrongo Health and Demographic Surveillance System. International Journal of Epidemiology. 2012; 41(4). DOI: 10.1093/ije/dys111 22933645

[28] VAST Study Team. Vitamin A supplementation in northern Ghana: effects on clinic attendances, hospital admissions, and child mortality. Lancet. 1993; 342(8862). DOI: 10.1016/0140-6736(93)91879-Q 8100345

[29] Kanmiki EW, Bawah AA, Agorinya I, et al. Socio-economic and demographic determinants of under-five mortality in rural northern Ghana. BMC International Health & Human Rights. 2014; 14. DOI: 10.1186/1472-698X-14-24 25145383 PMC4144693

[30] Kunutsor S, Powles J. Descriptive epidemiology of blood pressure in a rural adult population in northern Ghana. Rural Remote Health. 2009; 9(2). DOI: 10.22605/RRH1095 19508111

[31] Ramsay M, Crowther NJ, Agongo G, et al. Regional and sex-specific variation in BMI distribution in four sub-Saharan African countries: The H3Africa AWI-Gen study. Global Health Action. 2018; 11(sup2). DOI: 10.1080/16549716.2018.1556561 PMC6407581 30845902

[32] Agongo G, Nonterah EA, Debpuur C, et al. The burden of dyslipidaemia and factors associated with lipid levels among adults in rural northern Ghana: An AWI-Gen sub-study. PLoS One. 2018; 13(11). DOI: 10.1371/journal.pone.0206326 PMC6261546 30485283

[33] Dye JF, Schatz IM, Rosenberg BA, Coleman ST. Constant comparison method: A kaleidoscope of data. Qualitative Report. 2000; 4(1). DOI: 10.46743/2160-3715/2000.2090

[34] Hsieh H, Shannon SE. Three approaches to qualitative content analysis. Qualitative Health Research. 2015; 15(9). DOI: 10.1177/1049732305276687 16204405

[35] NVivo. Qualitative Data Analysis Software. GRS International; 2012.

[36] Khetan AK, Purushothaman R, Chami T, et al. The effectiveness of community health workers for CVD prevention in LMIC. Global Heart. 2017; 12(3). DOI: 10.1016/j.gheart.2016.07.001 27993594

[37] Ogedegbe G, Gyamfi J, Plange-Rhule J, et al. Task shifting interventions for cardiovascular risk reduction in low-income and middle-income countries: A systematic review of randomised controlled trials. BMJ Open. 2014; 4(10): e005983. DOI: 10.1136/bmjopen-2014-005983 PMC4202019 25324324

[38] Abdel-All M, Putica B, Praveen D, Abimbola S, Joshi R. Effectiveness of community health worker training programmes for cardiovascular disease management in low-income and middle-income countries: A systematic review. BMJ Open. 2017; 7(11): e015529. DOI: 10.1136/bmjopen-2016-015529 PMC5695434 29101131

[39] Labhardt ND, Balo JR, Ndam M, Grimm JJ, Manga E. Task shifting to non-physician clinicians for integrated management of hypertension and diabetes in rural Cameroon: A programme assessment at two years. BMC Health Service Research. 2010; 10. DOI: 10.1186/1472-6963-10-339 PMC3018451 21144064

[40] Erhun W, Agbani E, Bolaji E. Positive benefits of a pharmacist-managed hypertension clinic in Nigeria. Public Health. 2005; 119(7). DOI: 10.1016/j.puhe.2004.11.009 15990127

[41] Gyamfi, J, Allegrante J, Iwelunmor J, et al. Application of the consolidated framework for implementation research to examine nurses’ perception of the task shifting strategy for hypertension control trial in Ghana. BMC Health Service Research. 2020; 20. DOI: 10.1186/s12913-020-4912-5 PMC6990487 31996195

[42] Javadi D, Feldhaus I, Mancuso A, Ghaffar A. Applying systems thinking to task shifting for mental health using lay providers: a review of the evidence. Global Mental Health. 2017; 4. DOI: 10.1017/gmh.2017.15 PMC5719475 29230310

[43] Mutamba BB, van Ginneken N, Smith Paintain L, Wandiembe S, Schellenberg D. Roles and effectiveness of lay community health workers in the prevention of mental, neurological and substance use disorders in low and middle income countries: A systematic review. BMC Health Services Research. 2013; 13. DOI: 10.1186/1472-6963-13-412 24119375 PMC3852794

[44] Jørgensen TSH, Wium-Andersen MK, Jorgensen MB, Osler M. The impact of mental vulnerability on the relationship between cardiovascular disease and depression. European Psychiatry. 2020; 63(1). DOI: 10.1192/j.eurpsy.2020.20 PMC7315880 32093792

[45] Ademola A, Boima V, Odusola A, Agyekum F, Nwafor CE, Salako BL. Prevalence and determinants of depression among patients with hypertension: A cross-sectional comparison study in Ghana and Nigeria. Nigerian Journal of Clinical Practice. 2019; 22(4). DOI: 10.4103/njcp.njcp_351_18 30975963

